# Supplementary material for: Identification of photoperiod-induced specific miRNAs in the adrenal glands of Sunite sheep (Ovis aries)
Source: Front Vet Sci. 2022 Jul 22;9:888207. doi: 10.3389/fvets.2022.888207 (PMC9354845; doi:10.3389/fvets.2022.888207)
Supplement: Supplementary file 1 [file Data_Sheet_1.ZIP › Supplementary file/miRDeep2 file.zip!/miRDeep2 file/Know_Structure/SPLP42A3/oar-let-7i.pdf]

miRBase precursor : oar-let-7i
Total read count : 293989
oar-let-7i read count : 293918
remaining reads : 71

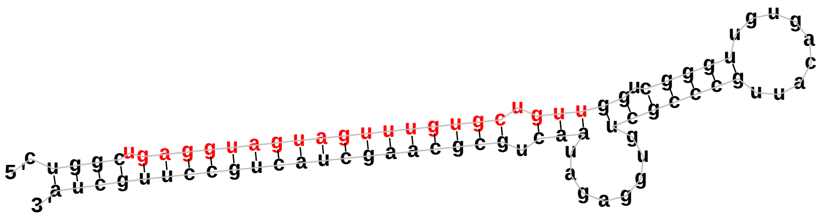

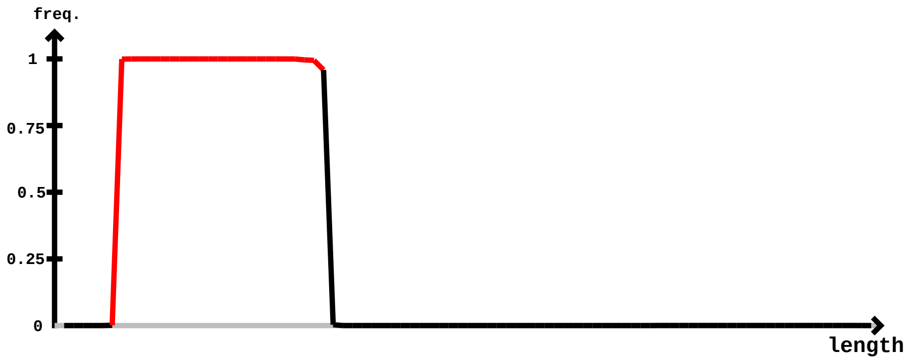


```
     oar-let-7i
5'- cuggcugagguaguaguuugugcuguuggucggguugugacauugcccgcuguggagauaacugcgcaagcuacugccuugcua   -3'   exp
    .((((.(((((((((((((((((.(((((.(((((.........)))))))........))).)))))))))))))))))))))    reads    mm    sample
    ....cugagguaguaguuugu...............................................................    1        0     seq
    ....cugagguaguaguuugugc.............................................................    1        0     seq
    ....cugagguaguaguuugugcu............................................................    3        0     seq
    ....cugagguaguaguuugugcug...........................................................    2        0     seq
    ....cugagguaguaguuugugcugu..........................................................    13       0     seq
    ....cugagguaguaguuugugcuguu.........................................................    317      0     seq
    ....cugagguaguaguuugugcuguug........................................................    1        0     seq
    .....ugagguaguaguuugug..............................................................    2        0     seq
    .....ugagguaguaguuugugG.............................................................    1        1     seq
    .....ugagguaguaguuugugc.............................................................    47       0     seq
    .....ugUgguaguaguuugugc.............................................................    1        1     seq
    .....ugagguaguaguuugugA.............................................................    6        1     seq
    .....ugagguaguaguuugugcu............................................................    952      0     seq
    .....ugagguaguaguuugugcug...........................................................    542      0     seq
    .....ugagguaguaguuugugcugu..........................................................    10551    0     seq
    .....ugagguaguaguuugugcuguu.........................................................    280380   0     seq
    .....ugagguaguaguuugugcuguug........................................................    938      0     seq
    .....ugagguaguaguuugugcuguugg.......................................................    3        0     seq
    .....ugagguaguaguuugugcuguuggu......................................................    1        0     seq
    ......gagguaguaguuugugcu............................................................    3        0     seq
    ......gagguaguaguuugugcugu..........................................................    8        0     seq
    ......gagguaguaguuugugcuguu.........................................................    140      0     seq
    ......gagguaguaguuugugcuguug........................................................    2        0     seq
    .......agguaguaguuugugcuguu.........................................................    3        0     seq
    .............................................................cugcgcaagcuacugccuug...    2        0     seq
    .............................................................cugcgcaagcuacugccuugc..    31       0     seq
    .............................................................cugcgcaagcuacugccuugcu.    36       0     seq
    .............................................................cugcgcaagcuacugccuugcua    2        0     seq
```
